# Pain and Health-Related Quality of Life in War Veterans with Bilateral Lower Limb Amputations

**DOI:** 10.5812/traumamon.5135

**Published:** 2012-07-31

**Authors:** Abolfazl Rahimi, Batool Mousavi, Mohammadreza Soroush, Mehdi Masumi, Ali Montazeri

**Affiliations:** 1 Faculty of Nursing, Baqiyatallah University of Medical Sciences, Tehran, IR Iran; 2 Department of Community and Preventive Medicine, Janbazan Medical and Engineering Research Center (JMERC), Tehran, IR Iran; 3 Janbazan Medical and Engineering Research Center (JMERC), Tehran, IR Iran; 4 Department of Public Health and Epidemiology, Mental Health Research Group, Health Metrics Research Centre, Iranian Institute for Health Sciences Research, Tehran, IR Iran

**Keywords:** Pain, Quality of Life, Lower Extremity, Amputation, Veterans

## Abstract

**Background:**

Amputation and pain may have considerable impact on health-related quality of life.

**Objectives:**

The purpose of this study was to assess the impact of pain on health-related quality of life in a population of war related bilateral lower limb amputees.

**Materials and Methods:**

The Veterans and Martyrs Affairs Foundation (VMAF) database documented 578 patients with bilateral lower limb amputation; 335 consented to the study (response rate = 58%). The majority of participants in the sample were males (96.7%). Types of pain were investigated using a questionnaire. Health-related quality of life (HRQOL) was investigated using the sf-36 questionnaire.

**Results:**

About two third of amputees reported phantom pain 66.7% (n = 223) and vertebral column pain 60.9% (n = 204). The most common type of pain was lumbosacral pain 52.8 % (n = 177) followed by neck 18.2 % (n = 61) and thoracic pain 9.6% (n = 32). Back pain affected on vitality, social function, mental health and mental component scale in our cases (P < 0.05). Neck pain affected all components of health-related quality of life (P < 0.05). Thoracic pain affected quality of life significantly (P < 0.05). The results obtained from logistic regression analysis indicated that none of the three spinal column pains including neck, thoracic and lumbosacral pain resulted in poor physical or mental component scales.

**Conclusions:**

This study revealed that bilateral lower limb amputees suffer from different types of pain and poor health-related quality of life. Therefore, the assessment and management of all types of pain are necessary to improve quality of life in veterans.

## 1. Background

Lower limb amputation is correlated to considerable morbidity, mortality and disability (1, 2). Research so far has shown that pain is a major risk factor of distress in a variety of cases. Many amputees experience significant amputation associated pain, which can affect their health-related quality of life (HRQOL) (3, 4), health, return to work (2), sleep and daily activities (5), and participation in social activities (6). Individuals with lower limb amputations often develop gait patterns to accommodate a prosthesis that may put them at risk for other types of pain such as hip and back pain and pain of the unaffected leg (7). Many studies have reported prevalence of pain types especially stump pain, phantom pain, phantom sensations (8, 9), and back pain after lower limb amputation (10-12).

Although the importance of prevention and health promotion in patients with lower limb amputation is well established (13), however, HRQOL in bilateral lower limb amputees has not been widely investigated (14). Additionally, information regarding the relationship between pain and quality of life in patients with loss of both lower limbs are more restricted (3). HRQOL in Iranian veterans with lower limb amputation has been previously evaluated and it has been shown that the war related bilateral lower amputees suffer from poor health related quality of life (15). However, there is no applicable evidence on the specific effect of pain on health-related quality of life in this group of veterans.

## 2. Objectives

The objective of this study was to assess the relationship between potential pain determinants and health-related quality of life in veterans with bilateral lower limb amputation.

## 3. Materials and Methods

### 3.1. Design and Data Collection

In a cross-sectional study, 578 veterans with bilateral lower limb amputation (either civilians or veterans) supported by the Veterans and Martyrs Affairs Foundation (VMAF) (16) were selected by census sampling. The inclusion criteria were: Being a war survivor, no underlying severe physical or mental condition, and willing to participate.

In order to collect data, interviews were conducted by 3 trained assessors. Each amputee was interviewed separately, face-to-face, for about 15-20 minutes. The amputees responded to demographic questions and were also asked about their amputation including the cause of the injury, amputation level and prosthesis usage. Information regarding history of neck, thoracic and lumbosacral pain. The participants were asked about bothersome and chronic pain in the three parts of the spinal column (cervical, thoracic and lumbosacral) in the past 6 months. The participants were also asked about phantom pain, its intensity and duration. Phantom pain intensity was rated as severe or not severe in one or both limbs (17). The data regarding duration of phantom pain was observed as always, usually, sometimes and rarely. The majority of participants in the sample were male (96.7%). All participants were informed about the purpose of the study and their participation was voluntary.

### 3.2. Quality of Life

Quality of life was measured using the 36-item Short Form Health Survey (SF-36). The SF-36 is a generic questionnaire that can be used for the general population and in different patient groups. The questionnaire consists of 36 questions that measure 8 health-related concepts. The SF-36 contains 8 domains; physical function (PF), physical role (PR), general health (GH), bodily pain (BP), vitality (VI), social functioning (SF), emotional role (RE), and mental health (MH). It also provides two summary scales: Physical Component Summary (PCS) and Mental Component Summary (MCS). Scores on each of the subscales range from 0 to 100, with 0 representing the worst health-related quality of life and 100 representing the best (18,19).

### 3.3. Statistical Analysis

In addition to descriptive statistics and independent t-test, we performed regression analysis to determine variables that mostly contributed to physical and mental health-related quality of life in patients with bilateral lower limb amputation. We consider a study power of 95% confidence interval and P- value less than 0.05 was significant. Data were analyzed by SPSS 16.0 statistical software.

### 3.4. Ethics

The ethics committee of Janbazan medical and engineering research center (JMERC), Tehran, I.R. Iran approved the study. All patients gave informed consent.

## 4. Result

Amputees ranged in age from 21 to 71 years (mean 42.05 yrs SD = 6.32 yrs). The average age of the amputees was 42.6 years (SD = 6.32). The average age at the time of injury was 22.6 years (SD = 4.3). The most common cause of amputation was related to blast injury due to grenades 56% (n=191) which was followed by land mine explosion 33% (n = 111) and bombing 4.7% (n = 16), respectively. Level of amputation in 37.9% (n = 124) of the cases was bilateral below knee, in 22.3% (n = 73) bilateral above knee, and in 34.3% (n = 112) of amputees level of amputation was a combination of above and below knee. Most of the cases used their prosthetics 80.3% (n = 269).

About two thirds of amputees 66.7% (n = 223) reported phantom pain. Of these 37.6% (n = 84) reported their pain to be severe, and 21% (n = 46) said that they always or usually suffered from phantom pain. There was a significant relationship between severity of phantom pain and three SF-36 domains including physical functioning, general health, and physical component scale (P < 0.05) Findings also showed a significant relationship between duration of phantom pain and physical functioning, bodily pain, mental health, and physical component scale (P < 0.05, Table 1).

**Table 1:** Comparison of the SF-36 Scores in Bilateral Lower Limb Amputees According to Phantom Pain Intensity and Duration

Phantom Pain Intensity ^a^			Phantom Pain Duration ^b^		
HRQOL Subscales	Mean (SD)	*P*	Duration	Mean (SD)	*P*
Physical Function		0.027			0.015
No	56.2 (22.6)		Usually	45.13 (20.49)	
Yes	48.7 (23.8)		Sometimes	54.51(23.46)	
Role-Physical		NS ^c^			NS
No	50.3 (24.9)		Usually	43.63 (21.75)	
Yes	45.8 (25.1)		Sometimes	49.36 (25.26)	
Bodily Pain		NS			0.025
No	47.1(22.2)		Usually	38.03 (20.21)	
Yes	41.4(20.8)		Sometimes	46.39 (22.66)	
General Health		0.017			NS
No	55.3 (26.5)		Usually	45.38 (23.30)	
Yes	48.1 (26.2)		Sometimes	53.89 (26.75)	
Vitality		NS			NS
No	63.1 (24.2)		Usually	56.42 (23.35)	
Yes	58.9 (23.2)		Sometimes	62.36 (23.95)	
Social Function		NS			NS
No	63.2 (26.1)		Usually	62.89 (28.64)	
Yes	65.9 (28.4)		Sometimes	64.11 (26.23)	
Role-Emotional		NS			NS
No	65.2 (26.6)		Usually	55.43 (24.27)	
Yes	59.7 (26.3)		Sometimes	63.63 (26.76)	
Mental Health		NS			0.016
No	63.1 (26.5)		Usually	51.96 (23.95)	
Yes	55.7 (24.5)		Sometimes	62.31 (25.97)	
Physical Component Scale		0.019	(n=117)		0.028
No	47.4 (21.1)		Usually	38.07 (17.09)	
Yes	40.2 (20.7)		Sometimes	45.7 (21.5)	
Mental Component Scale		NS			NS
No	58.3 (25.8)		Usually	51.03 (22.44)	
Yes	54.1 (24.5)		Sometimes	57.57 (25.52)	

^a^Severe (Yes) (n = 84) Not severe (No) (n = 139)

^b^Usually (n = 46) Sometimes (n = 117)

^c^Abbreviation: NS: Not Significant

About two thirds of the amputees 60.9% (n = 204) reported vertebral column pain. The most common type of pain was lumbosacral pain 52.8 % (n = 177) followed by neck 18.2 % (n = 61) and thoracic pain 9.6% (n = 32). Existence of vertebral pain was significantly associated with lower scores in bodily pain (BP) (P < 0.009), vitality (VI) (P < 0.001), social functioning (SF) (P < 0.01), mental health (MH) (P < 0.003), physical component scale (P < 0.02) and mental component scale (P < 0.002).

Neck pain affected on all the components of health related quality of life except physical and emotional components. The subject who suffered from thoracic pain had significantly lower scores in physical function (PF), general health (GH), vitality, social function, mental health, physical component scale and mental component scale. Having back pain was also significantly associated with lower scores in bodily pain, vitality, social function, mental health and mental component scale (Table 2).

**Table 2:** Comparison of the SF-36 Scores in Bilateral Lower Limb Amputees for Vertebral Pain

	Neck Pain Mean (SD)			Thoracic Pain Mean (SD)			Lumbosacral Pain Mean (SD)		
HRQOL Subscales	Yes (n = 61)	No (n = 274)	*P*	Yes (n = 28)	No (n = 307)	*P*	Yes (n = 117)	No (n = 158)	*P*
Physical function	48.0 (23.4)	56 (24.5)	0.02	41 (24.9)	55.7 (24.1)	0.002	53 (24.9)	56.2 (24)	NS
Role physical	45.6 (22.3)	51(25.6)	NS^a^	44 ( 21)	50.6 (25.3)	NS	48.7 (24.7)	51.6 (25.5)	NS
Bodily pain	41.2 (22.7)	49.5 (24.1)	0.01	41.9 (19.8)	48.5 (24.3)	NS	45.4 (24)	50.9 (23.8)	0.03
General health	45.6 (26.9)	57.4 (263)	0.002	41.6 (24.9)	56.6 (26.6)	0.004	54.3 (26)	56.5 (27.6)	NS
Vitality	57 (23.1)	64.9 (23.6)	0.01	52 (21.1)	64.5 (23.7)	0.007	59.4 (24)	68 (23.2)	0.001
Social function	60.4 (26.4)	68.1 (26.7)	0.04	51.5 (30)	68.1 (26.1)	0.002	63.5 (27.2)	70.2 (25.8)	0.02
Role emotional	59.3(24.8)	64 (27.2)	NS	56.3(24.3)	63.8(27)	NS	60.8(26.2)	65.7(27.2)	NS
Mental health	53.6 (25)	64.5 (25.1)	0.002	46.6 (25)	64 (25)	0.001	59 (25.3)	66.5 (24.9)	0.006
Physical component scale	37.9 (21.6)	48.7 (22.3)	0.001	31.8 (18.7)	48.1 (22.4)	0.001	44.9 (22)	48.9 (23.1)	NS
Mental component scale	51.1 (23.5)	60.3 (25.4)	0.01	44.8 (23.4)	59.9 (25.1)	0.002	54.9 (24.5)	63 (25.6)	0.003

^a^Abbreviation: NS: Not Significant

## 5. Discussion

This study assessed impact of pain on HRQOL in Iranian survivors of the Iraq-Iran war with bilateral lower limb amputation. Poor QOL was the most important finding of this study. In this regard a study by Dajpratham et al (2011) showed that Thai people with unilateral LLA reported primarily fair HRQOL and the number of people with LLA who had fair QOL were more than eighty six percent (20). Also our study indicates that amputees who reported to have vertebral column or phantom pain had a considerable poorer HRQOL comparing with their counterparts.

Pain has a considerable impact on HRQOL (3). Individuals with lower limb amputations often develop gait patterns to accommodate a prosthesis that may put them at risk for other types of pain such as back pain (10). Frequency of back pain among lower limb amputees varies from 47.7 to 94.7% in different studies (10-12), in our study more than half of the participants reported back pain. In the study by Hammarlund et al. (2011) there was a high prevalence of back pain after amputation (21), which is consistent with the findings of our study.

Lower limb amputation commonly leads to asymmetrical weight-bearing, even after rehabilitation treatment and in particular leads to additional weight gain which causes back and extremity pain. This is detrimental to the amputees’ long-term HRQOL (22). Some risk factors for back pain such as work-related factors, educational level, exercise and individual factors, directly or indirectly affect SF-36 scores in individuals and need consideration in the evaluation and interpretation of the HRQOL (23). Study of Asano et al. (2008) revealed some other significant factors as predictors of subjects’ perceived QOL in lower limb amputation including: depression, perceived prosthetic mobility, social support, co-morbidity, prosthesis problems and social activity participation. Several modifiable characteristics influence QOL after lower limb amputation including depression and participation in daily living. These findings suggest the importance of addressing individuals’ perception status to regain or maintain QOL (24).

Prevalence of lumbosacral pain was higher than other pains in the amputees. Similar results have been shown by Taghipour et al. (2009) study, in which poor physical HRQOL was positively associated with transfemoral amputation, phantom movement and back pain (25). The results of this study showed that neck pain compared to other types of pain was more accompanied with lower scores on SF-36 domains especially on physical function, general health, bodily pain, vitality, social function and mental health in our subjects.

The prevalence of phantom pain was also high in our study population. This result is consistent with the study of Wartan et al. (26) and is somewhat lower than the prevalence reported by Sherman et al. (5), and Dijkstra et al. (8). Our study also showed that amputees with phantom pain suffered more from poor HRQOL compared to their counterparts. This finding is consistent with the findings of Van der Schans et al. (2002) (3) and Lerner et al. (27). Because of the high reported prevalence and the impact of phantom pain on medical care and quality of life, identification of risk factors associated for phantom pain may be important (8).

As shown in Table 2 amputees with severe phantom pain had poorer quality of life in physical health subscales more than mental health subscales. Similar results were found by Mousavi et al. (2009) that victims scored better on mental health related subscales than physical health (16). Moreover, amputees who reported that their phantom pain is usually or always severe, had lower scores in both physical and mental component subscales of SF-36. Back Pain and other types of pain are common after limb loss (22, 23, 25). In this survey we did not study intensity, quality and duration of spinal pain. Further research is recommended to focus on these topics. According to results of this study, it can be concluded that pain in BLLA was accompanied by poor health-related quality of life. This situation remarks an indication that patients need more support from the healthcare system. The recognition of risk factors contributing to pain and health-related quality of life may result in the development of interventions aimed at the prevention or treatment and improvement of HRQOL in war veterans.
